# Isolated Radial Vein Thrombosis: Upper Extremity Deep Vein Thrombosis in a Patient With COVID-19 Infection

**DOI:** 10.7759/cureus.12856

**Published:** 2021-01-22

**Authors:** Sathishkumar Ramalingam, Harkesh Arora, Kulothungan Gunasekaran, Maheswari Muruganandam, Sivakumar Nagaraju

**Affiliations:** 1 Hospital Medicine, Lovelace Medical Center, Albuquerque, USA; 2 Pulmonary Critical Care, Yale New Haven Health Bridgeport Hospital, Bridgeport, USA; 3 Rheumatology, University of New Mexico, Albuquerque, USA; 4 Pulmonary Critical Care, Lovelace Medical Center, Albuquerque, USA

**Keywords:** radial vein thrombosis, covid coagulopathy, covid-19, upper extremity dvt, deep vein thrombosis (dvt), upper extremity thrombosis

## Abstract

In general, upper extremity deep vein thrombosis (DVT) is less common than lower extremity DVT. Among upper extremity DVT cases, most of them are due to secondary causes like indwelling catheters, cancer, surgery, trauma or immobilization by plaster casts, pregnancy, oral contraceptives, and estrogen. Patients with coronavirus disease 2019 (COVID-19) infection are known to have coagulation dysfunction and a high incidence of DVT, mostly in the lower extremities; however, upper extremity DVT has been rarely reported. We present a rare case of upper extremity DVT in COVID-19 infection. A 56-year-old male with no significant past medical history was admitted with acute respiratory failure due to COVID-19 pneumonia. During hospitalization, he developed right upper extremity swelling, and an ultrasonogram showed right radial vein thrombosis. He was initially started on low molecular weight heparin (LMWH) and was discharged on apixaban. Patients with COVID-19 infection who develop DVT are recommended treatment with a direct oral anticoagulant (DOAC) for three months.

## Introduction

Deep vein thrombosis (DVT) is a complication of hospitalized patients due to less mobility and other underlying medical illness [1]. Patients with coronavirus disease 2019 (COVID-19) infection have a high incidence of DVT as they have coagulation abnormalities [2]. While upper extremity DVT is less common than lower extremity DVT, an isolated radial vein DVT is an extremely rare presentation. We present a case of a 56-year-old male admitted with acute respiratory failure due to COVID-19 infection and was found to have right radial vein thrombosis.

## Case presentation

**Figure 1:**
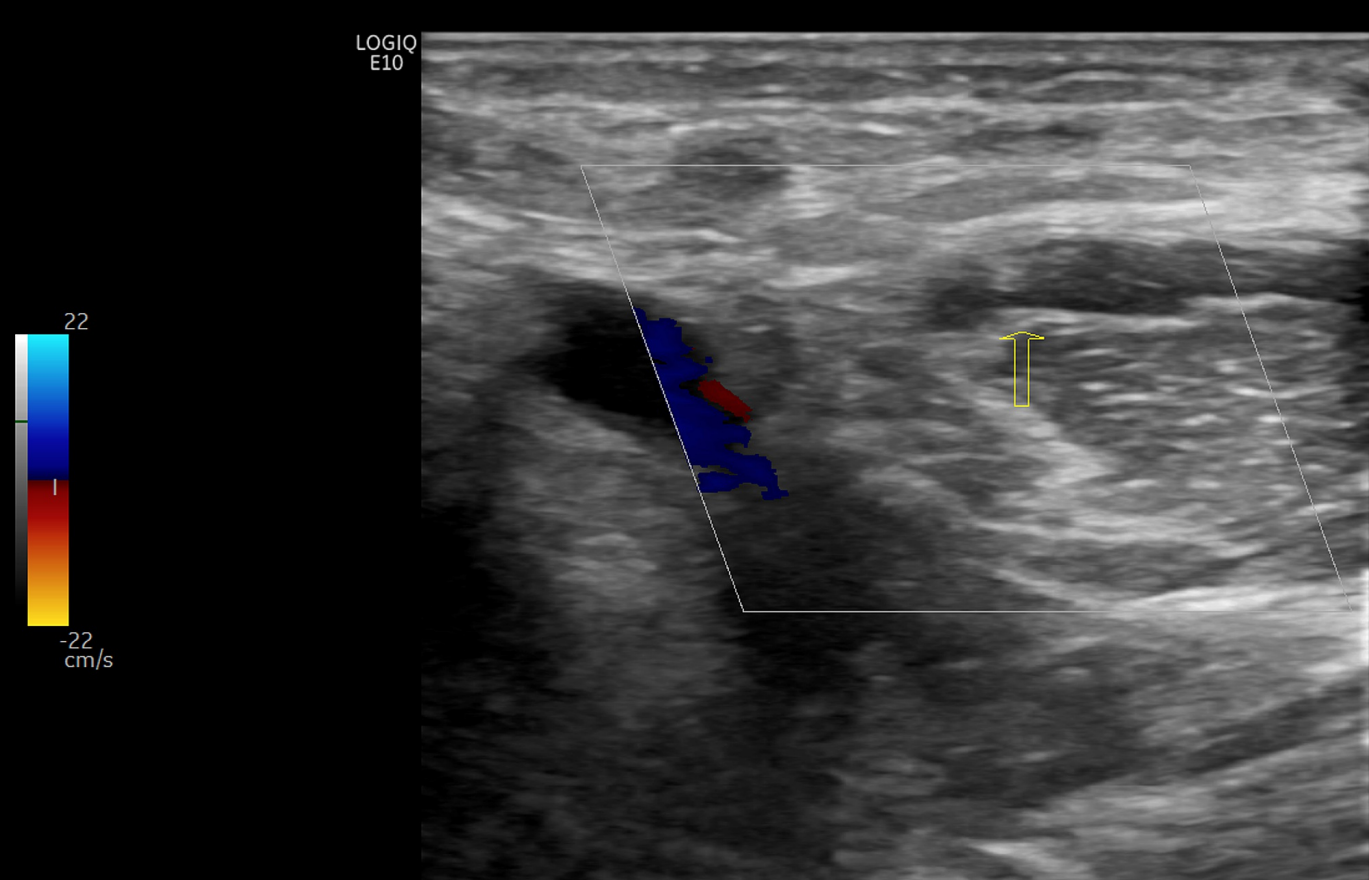
Venous duplex of the bilateral upper extremities Deep vein thrombus noted within the right proximal radial vein.

A 56-year-old male with no significant past medical history presented with shortness of breath, cough, headache, and body pain for four days. His temperature was 98°F, blood pressure was 142/91 mmHg, heart rate 92/min, and oxygen saturation was 88% on room air. His white blood cell was 5.1 K/uL, platelet 122 K/uL, D-Dimer was 476 ng/mL, ferritin 2184 ng/mL, erythrocyte sedimentation rate (ESR) 45 mm/hr, and C-reactive protein (CRP) 27 mg/dL. Chest X-ray revealed bilateral infiltrates. Reverse transcription-polymerase chain reaction detected severe acute respiratory syndrome coronavirus 2 (SARS-CoV-2) via a nasopharyngeal swab and he was admitted to the hospital. Peripheral venous access was obtained on the left forearm and was he started on remdesivir and dexamethasone. He was placed on low molecular weight heparin (LMWH) 0.5 mg/kg every 12 hours for DVT prophylaxis. He developed acute respiratory failure requiring 15 L of oxygen and did not require high flow oxygen or non-invasive positive pressure ventilation (NIPPV). On day six of hospitalization, he developed right upper extremity swelling, and D-Dimer was 4674 ng/dL. Bilateral venous Doppler ultrasound of the upper extremity showed acute DVT within the right proximal radial vein (Figure 1). Lower extremity doppler ultrasound was unremarkable for any DVT. Computed tomography (CT) scan of the chest with contrast showed bilateral ground-glass opacities and no pulmonary embolism (PE). He was started on 1 mg/kg of enoxaparin (therapeutic dose) every 12 hours, subsequently switched to oral apixaban. His oxygen demand improved, and he was discharged home with 3 liters of oxygen and apixaban.

## Discussion

DVT is one of the complications of hospitalized patients who are bedridden with multiple medical problems. Upper extremity DVT is less common but still accounts for 5%-10% of all DVT cases, mostly associated with the intravenous catheter [3]. The most common anticoagulants used for DVT prophylaxis are enoxaparin and heparin. Mechanical prophylaxis includes graduated compression stockings (GCS) and intermittent pneumatic compression (IPC) devices [4]. Coagulation abnormalities have been noted in patients with COVID-19, and their exact mechanism is not well understood. The role of COVID-19 on the angiotensin-converting enzyme 2 receptors on endothelial cells and cytokine storm has been found to play a role in thromboembolic complications [5]. Patients with COVID-19 infection with increased D-Dimer, low platelets, and prolonged prothrombin time on initial presentation have a high risk of developing venous thromboembolism (VTE) and disseminated intravascular coagulation (DIC) [6,7]. Thrombotic events, including PE, DVT, myocardial infarction (MI), and a cerebrovascular accident, have been reported in patients with COVID-19 infection [8,9]. There are only a few upper extremity DVT reported in patients with COVID-19 infection [10]. Isolated radial vein thrombosis is an exceedingly rare presentation, and no case has been reported in patients with COVID-19 infection. Patients hospitalized with COVID-19 infection have a high risk of developing VTE and should be started on standard anticoagulation thromboprophylaxis (LMWH every 12 hours). Our patient developed DVT despite being on prophylactic anticoagulation. Patients with proximal DVT need to be started on LMWH or intravenous unfractionated heparin or direct oral anticoagulants (DOAC) like apixaban or rivaroxaban [11]. Patients with upper extremity DVT involving proximal vein should be treated with oral anticoagulation, and there is no benefit from thrombolytic therapy. The recommended treatment and duration for a patient with upper extremity DVT is a DOAC for at least three months unless otherwise contraindicated [12].

## Conclusions

The incidence of VTE in COVID-19 patients is higher than in the general population, even if they receive prophylactic anticoagulation. There must be high suspicion of DVT in patients with COVID-19 as timely treatment with anticoagulation can prevent life-threatening PE. Even though upper extremity DVT is less common, clinicians should be vigilant in screening COVID-19 patients as there will be significant comorbidities associated with it.
